# Supplementary material for: Measuring the conservation attitudes of local communities towards the African elephant Loxodonta africana, a flagship species in the Mara ecosystem
Source: PLoS One. 2021 Jun 23;16(6):e0253234. doi: 10.1371/journal.pone.0253234 (PMC8221505; doi:10.1371/journal.pone.0253234)
Supplement: S1 Appendix — (DOCX) [file pone.0253234.s001.docx]

**S1 Appendix 1**

**Household Questionnaire**

**Form No._________**

**NAME OF INTERVIEWER ________________VILLAGE/ AREA _____________________________**

**DATE (D/M/Y): ____________________________GPS X🗌🗌🗌🗌🗌🗌🗌 Y🗌🗌🗌🗌🗌🗌🗌**

**START TIME: __________ ____________________END TIME: _____________________________________**

**NAME OF RESPONDENT __________________________________________________________________**

The purpose of this questionnaire is to understand how the local residents in Trans Mara interact with wildlife and utilize their environment to meet their livelihood needs. Any information you provide **will** be used anonymously. You do not have to answer any question you do not feel comfortable with and you can stop the interview process at any time. You can ask for clarification on any question at any time. This questionnaire is part of an independent study being conducted by Tobias Nyumba, a PhD student from The University of Cambridge in the U.K. We would be very grateful if you could participate. Thank you for your cooperation.

1. **GENERAL INFORMATION**

| **Code** | | **Age *(years)*** | **Sex**  ***1. M 2. F*** | **Relationship to household head** | **Education Level** | **Ethnicity** | **Marital status** |
| --- | --- | --- | --- | --- | --- | --- | --- |
| 1. 1 | Respondent |  |  |  |  |  |  |
|  |  |  |  |  |  |  |  |
| 1. 2 |  |  |  |  |  |  |  |
| 1. 3 |  |  |  |  |  |  |  |

1. **We are interested in learning about the people who live in your household. Can you please tell us the following about all members of your household?**
2. **Do you live here most of the time?** **🗌**=Yes **🗌**=No

**If NO, where do you live most of the time?**____________________________________

1. **HUMAN RESOURCES**
2. **Can you please tell us the two main activities/occupations of household members in the last 12 months?** *(Use the table below for reference)*

| **Code** | **Main activities/ occupations** | | **What kind of activities are these?** | | **Where were these activities/occupations conducted?** | | **Do these main activities/occupations significantly vary by seasons?**  ***1…Yes 2…No*** |
| --- | --- | --- | --- | --- | --- | --- | --- |
|  | **First** | **Second** | **First** | **Second** | **First** | **Second** |  |
| 1 |  |  |  |  |  |  |  |
| 2 |  |  |  |  |  |  |  |

| **Mani Activity/Occupation** | | **Main Activity/Occupation** | | **Kind of Activity/Occupation** | |
| --- | --- | --- | --- | --- | --- |
| *Agricultural* | | *Professional* | |  |  |
| 1 | Farmer | 14 | Administrative/Clerical | 1 | Self Employed |
| 2 | Farm laborer | 15 | Doctor | 2 | Unpaid family work |
| 3 | Herder | 16 | Health worker | 3 | Regular work for cash |
| *Artisanal* | | 17 | NGO Worker | 4 | Occasional work for cash |
| 4 | Carpenter | 18 | Religious worker | 5 | Daily wage labor |
| 5 | Cobbler | 19 | Soldier | 6 | Food for work |
| 6 | Hand crafts | 20 | Teacher | 7 | Community work |
| 7 | Goldsmith | *Home Worker* | | 8 | Education/training |
| *Non-Agricultural* | | 21 | Housewife | 9 | Non-work activities |
| 8 | Construction laborer | 22 | House help |  |  |
| 9 | Manual worker | *Commercial* | |  |  |
| 10 | Factory/mill worker-Skilled | 23 | Business |  |  |
| 11 | Factory/mill worker-unskilled | 24 | Hotel |  |  |
| *Transport* | | 25 | Petty trader |  |  |
| 12 | Mechanic | *Not in labor force* | |  |  |
| 13 | Driver | 26 | Studying |  |  |
|  |  | 27 | Disabled/Sick |  |  |

1. **Can you please tell us about the education of household members that are currently attending an educational facility?**

| **Code** | **What is the level of education so far?** | **What type of educational facility is it?** | **Where is the educational facility?** | **What mode of transport is used to go to the educational facility?** | **Approximately how long does it take to reach the educational facility (one way)?*** |
| --- | --- | --- | --- | --- | --- |
| 1 |  |  |  |  |  |
| 2 |  |  |  |  |  |

**1=Less than 30mins; 2= 30-60mins; 3=1-2hrs; 4=More than 2hrs; 5= Attending boarding school*

1. **Can you please tell us about the illness and injuries of all household members?** *(Use the table below for reference)*

| **Code** | **Does the person suffer from chronic ill health?**  ***1. Yes 2. No*** | **Chronic ill health**  ***If no chronic ill health, write 0.*** | **Does the person suffer from major disability?**  **1*. Yes 2 1. No*** | **Major disability**  ***If no major disability, write 0.*** | **In the last 12 months has the person been so ill/injured that s/he was unable to perform usual Daily activities?**  ***1.Yes 2. No*** | **What was the illness/injury?** | **How long did this period of illness/injury last?** |
| --- | --- | --- | --- | --- | --- | --- | --- |
|  |  |  |  |  |  |  |  |
| 1 |  |  |  |  |  |  |  |
| 2 |  |  |  |  |  |  |  |

| ***Chronic ill health*** | | ***Major disability*** | | ***How long?*** | | |
| --- | --- | --- | --- | --- | --- | --- |
| *1* | *Fever, sweating* | *1* | *Vision* | *1* | | *Less than a week* |
| *2* | *Dehydration* | *2* | *Hearing* | | *2* | *About a week* |
| *3* | *Diarrhoea* | *3* | *Appearance* | | *3* | *About two weeks* |
| *4* | *Malnutrition* | *4* | *Other sensory* | | *4* | *Up to a month* |
| *5* | *Under/over weight* | *5* | *Poor muscle control* | | *5* | *More than a month* |

**3. MATERIAL REOSURCES**

1. **Does the household own, have access or no access to the following assets?**

| **Type** | **Own** | **Access** | **No Access** |
| --- | --- | --- | --- |
| Car/Pickup |  |  |  |
| Motorcycle |  |  |  |
| Bicycle |  |  |  |
| Tractor |  |  |  |
| Plough |  |  |  |
| Water storage tank |  |  |  |
| Pit latrine |  |  |  |
| Radio |  |  |  |
| Television |  |  |  |
| Cell phone |  |  |  |
| Solar Panel |  |  |  |
| Electricity |  |  |  |
| Other (Please Specify) |  |  |  |

1. **Does the household own any livestock? 🗌**=Yes **🗌**=No

| **Type** | **Yes** | **No** | **Number** | **Visual Verification (Please Tick)** |
| --- | --- | --- | --- | --- |
| Cattle | 1 | 2 |  |  |
| Goats | 1 | 2 |  |  |
| Sheep | 1 | 2 |  |  |
| Chickens | 1 | 2 |  |  |
| Donkeys | 1 | 2 |  |  |
| Other______________ | 1 | 2 |  |  |

1. **Which of the following is true about the household?**

Owns all the land it uses **🗌**

Own some of the land it uses **🗌**

Rent in land from others **🗌**

Rent out land to others **🗌**

Borrow land from others **🗌**

Lease land from community on long-term contract **🗌**

Lease land from community on short-term contract **🗌**

Use land through other arrangements (Please Specify) **🗌**

1. **What other natural resources does the household use for consumption, production or sale?**

Water (rivers, ponds, streams) For drinking **🗌**

For Irrigation **🗌**

For fishing **🗌**

Livestock watering **🗌**

Other household uses **🗌**

Land For mining **🗌**

For grazing **🗌**

For wild crop harvesting **🗌**

Trees For timber **🗌**

For firewood **🗌**

For fruits **🗌**

For medicine  **🗌**

1. **What is the quality of the respondent’s house like?** *(Interviewer to make this assessment)*

Below standard 🗌 Standard 🗌 above standard 🗌

1. **Does the household own the house?**

Yes 🗌 No 🗌

1. **During the last 12 months, did your household suffer any shortages of staple foods (cereals, etc)/vegetable proteins (peas, beans)/fruits/or animal proteins (eggs, dairy products, meat) for more than 1 month?**

**Yes No**

Staple foods 🗌 🗌

Vegetables/fruits 🗌 🗌

Vegetable proteins 🗌 🗌

Animal proteins 🗌 🗌

1. **SOCIAL RESOURCES**

| **Code** | **Type of Organization** | **Name of organization** | **Type of Participation** | **Type of second organization** | **Name of second organization** | **Type of participation in Second organization** |
| --- | --- | --- | --- | --- | --- | --- |
| 1 |  |  |  |  |  |  |
| 2 |  |  |  |  |  |  |

1. **In the last 12 months, have you or any other household member been a member of any club, association, society, cooperative or other forms of organisation in the community?** *(Use the table below for reference)*
2. **In the last 12 months, have you or any other household member participated in any form of collective community activity?** *(Use the table below for reference)*

| **Code** | **Type of Activity** | **Name of Activity** | **Type of Participation** |
| --- | --- | --- | --- |
| 1 |  |  |  |
| 2 |  |  |  |

| ***Type of organization*** | | ***Type of activity*** | | | ***Type of participation*** | |
| --- | --- | --- | --- | --- | --- | --- |
| *1* | *Community based* | *1* | | *Economic* | *1* | *Leader* |
| *2* | *External-Govt* | *2* | *Political* | | *2* | *Manager* |
| *3* | *External-NGO* | *3* | *Social and Cultural* | | *3* | *EC Member* |
| *4* | *Other* | *4* | *Other* | | *4* | *Active member* |
|  |  |  |  | | *5* | *General member* |

1. **If you or any member of your household wanted to know about events in the capital city or elsewhere in the country, which of the following would you do?**

Listen to Radio 🗌

Watch TV 🗌

Read newspaper 🗌

Write a letter 🗌

Talk on telephone 🗌

Ask someone who has been travelling outside the community 🗌

Attend a local event or rally 🗌

1. **In terms of quality and quantity, how have government support programmes been in your village in the last 12 months?**

🗌 Poor 🗌 Reasonable 🗌 Good

1. **In terms of quality and quantity, how have non-governmental support programmes (NGOs, Conservancies, CBOs) been in your village in the last 12 months?**

🗌 Poor 🗌 Reasonable 🗌 Good

1. **INCOME AND EXPENDITURE**
2. **What are the things that you and other members of your household did to make money in 2014? Please list all activities and what was earned from each in 2014. Also, please rank their importance to the household, with “1” being most important and “6” the least important**

| **Source of Income** | **Rank** | **Value (Ksh)** |
| --- | --- | --- |
| Livestock Sales |  |  |
| Employment |  |  |
| Farm produce |  |  |
| Craft sales |  |  |
| Natural products (Firewood/Charcoal/poles/etc) |  |  |
| Other (Please Specify) |  |  |

1. **Are the household’s sources of income regular?**

Yes, most income sources are regular 🗌

No regular income source 🗌

1. **If products were sold, who purchased them?**

| **Product** | **Buyer** | **Sale Value (Ksh)** |
| --- | --- | --- |
| Livestock Sales |  |  |
| Cash crops |  |  |
| Firewood |  |  |
| Medicinal plants |  |  |
| Natural products (Firewood/Charcoal/poles/etc) |  |  |
| Other (Please Specify) |  |  |

1. **Please list all your household expenses in the last one year in the following categories. Please rank their importance in your household with “1” being the most important and “7” the least important**
2. **WELLBEING ASSESSMENT**

| **Expense Category** | **Approximate amount (Ksh)** | **Time frame (Per year/Per month)** | **Rank** |
| --- | --- | --- | --- |
| Food |  |  |  |
| School |  |  |  |
| Transport |  |  |  |
| Medical care |  |  |  |
| Clothes |  |  |  |
| Labour |  |  |  |
| Household electronics/phones |  |  |  |

1. **Is your household prosperous?**  🗌 No, it is not prosperous 🗌 Fairly 🗌 Yes, it is prosperous
2. **Have there been any shortages of food for more than 1 month during the past 12 months?**  🗌 Yes 🗌 No
3. **Concerning your family’s food consumption over the past one month, which of the following is true? The family’s food consumption was**

🗌 Not adequate 🗌Just adequate 🗌More than adequate

1. **Concerning your family’s clothing, which of the following is true? The family’s clothing is** 🗌 Not adequate 🗌Just adequate 🗌More than adequate
2. **Where does your household get drinking water?**

Public Tap 🗌

Well/borehole (also used by livestock) 🗌

Well/borehole not used by livestock 🗌

River (same location as where livestock goes) 🗌

River (different location as where livestock goes) 🗌

Other (Please Specify) 🗌

1. **Is the drinking water that you have access to clean?**

🗌 Yes, always 🗌 Yes, but only sometimes 🗌 No

1. **What is the quality of water like in nearest river?**

🗌 Bad 🗌Reasonable 🗌Good 🗌Don’t Know

1. **Has drinking water become easier or harder to obtain in recent years?**

🗌 Easier 🗌 Harder 🗌 Remained the same. Explain____________________

1. **How are feelings of mutual trust among community members in the community?**

🗌Low 🗌Medium 🗌High

1. **Do conflicts arise between people or families in the community?**

🗌Yes, frequently 🗌 Sometimes 🗌 Rarely occur

1. **In the event of sickness, do members of your household always receive modern medical treatment from a doctor, nurse, midwife, or traditional care from a healer?**

🗌 Never 🗌 Sometimes 🗌 Yes, always 🗌 Nobody has been sick

1. **How much of the natural environment (e.g. forest, grassland, river) around your village is damaged?**

🗌Half or more 🗌 Less than Half 🗌None 🗌Don’t Know

1. **How difficult is it to get to the nearest healthcare facility (dispensary, community health centre, hospital, etc.)?**

🗌 Very difficult / impossible 🗌 Difficult, but usually possible 🗌 Easy

1. **Concerning the health care your family gets which of the following is true? The health care your family get is**

🗌 Not adequate 🗌Just adequate 🗌More than adequate 🗌Not applicable

1. **Do you consider your household to be poor?** 🗌 Yes, it is poor 🗌 Fairly 🗌 No

1. **Concerning your family’s housing which of the following is true? The family’s housing is** 🗌 Not adequate 🗌Just adequate 🗌More than adequate
2. **How difficult is it to get to the nearest primary school?**

🗌 Very difficult / impossible 🗌 Difficult, but usually possible 🗌 Easy

1. **Concerning your children’s education which of the following is true? The education they get is** 🗌 Not adequate 🗌Just adequate 🗌More than adequate 🗌Not applicable
2. **How difficult is it to get to the nearest market?**

🗌 Very difficult / impossible 🗌 Difficult, but usually possible 🗌 Easy

1. **In what condition are the roads and bridges leading to the nearest market centre?**

🗌 There are none 🗌 In bad repair 🗌 In good condition

1. **SUBJECTIVE QUALITY OF LIFE ASSESSMENT**

The following questions will ask how **HAPPY** you feel with different aspects of your life. On a 5-point scale, you may feel:

*1 ☹☹ Very unhappy*

*2 ☹ Unhappy*

*3 😐 Neutral: Neither happy nor unhappy*

*4 ☺ Happy*

*5 ☺☺ Very happy*

1. **Thinking about your own life and personal circumstances, how do you feel about each of the following aspects?**
2. **Over the past ten years, have your feelings changed (More happy now or 🡽; Less happy now🡾 or Stayed the same ⬄)**
3. **Explain how your feelings have changed.**

|  | 1. **Very unhappy-Very Happy** | | | | | 1. **Has it changed?** | | | 1. **Why has it changed?** |
| --- | --- | --- | --- | --- | --- | --- | --- | --- | --- |
|  | *☹☹* | *☹* | *😐* | *☺* | *☺☺* |  |  |  |  |
|  |  |  |  |  |  | 🡽 | ⬄ | 🡾 |  |
| Your life as whole |  |  |  |  |  |  |  |  |  |
| Your shelter and living conditions |  |  |  |  |  |  |  |  |  |
| Your own health |  |  |  |  |  |  |  |  |  |
| Your achieving in your life |  |  |  |  |  |  |  |  |  |
| Your personal relationships (Marriage, communication with friends and family) |  |  |  |  |  |  |  |  |  |
| Your personal safety |  |  |  |  |  |  |  |  |  |
| Being a member of the village |  |  |  |  |  |  |  |  |  |
| Your future security (having access to land, good crops, freedom, education) |  |  |  |  |  |  |  |  |  |
| Availability of water for livestock and crops |  |  |  |  |  |  |  |  |  |

Remarks___________________________________________________________________________________________

**8. INTERRACTION WITH ELEPHANTS**

1. **In the last year, have you seen elephants in this area?** 🗌 Yes 🗌 No

**If yes in which months?** _____________________________________________________________

1. **When did you last see elephants in this area?**

🗌In the last week 🗌In the last month

🗌In the last three months 🗌In the last six months

🗌Longer than six months ago 🗌Other (Please Specify)_________________________

1. **Where were they when you noticed them?**

🗌Near my homestead 🗌In a field

🗌In the forest 🗌In the MMNR

🗌In the community conservancy 🗌Other (Please Specify)_______________________

1. **What were you doing when you noticed them?**

🗌Travelling on foot/bicycle 🗌travelling by motorbike/vehicle

🗌Tending crops 🗌looking after livestock

🗌Collecting wild foods 🗌fetching water

🗌Collecting firewood 🗌other (Please Specify)_______________________

1. **What time of day did you notice them?**  🗌Night 🗌Day
2. **How many were there?**

🗌Don’t know 🗌only 1 🗌1-5 🗌5-15 🗌15-25 🗌25-50 🗌>50

1. **Do you grow crops?** 🗌 Yes 🗌 No

**If Yes (a) why do you grow crops?** 🗌Commercial 🗌Subsistence 🗌Both

**(b) When did you start farming? __________________________________________**

**(c) How much land is under cultivation (in Hectares)**

🗌<1 🗌1-2 🗌2-4 🗌5-10 🗌>10

**If NO, (a) Why? _____________________________________________________________________**

**(b) Do you intend to start farming in the near future?** 🗌 Yes 🗌 No

1. **Did you have any problems with crop raiding in 2014?** 🗌 Yes 🗌 No

**If Yes, how much did you lose?**

🗌 All 🗌 More than half 🗌Less than half 🗌 Almost nothing

1. **How do you prevent elephants from destroying your crops?**

🗌Guarding farms 🗌Lighting fires 🗌 Moats and trenches 🗌Torch

🗌Thunder flashes 🗌 Chilli dung smoke 🗌Home-made bangers

🗌Noise *(shouting, drum beating)* 🗌Watchtower and spotlight 🗌Fence_____________________________________________________

🗌Other_____________________________________________________

1. **Has crop raiding increased or decreased since you can remember?**

🗌Increased 🗌Decreased 🗌 Stayed the same 🗌Don’t know

1. **Besides crop raiding, what other problems do you encounter with elephants?**
2. **Has any of your family member or neighbour been** 🗌 Killed 🗌 Injured

🗌 Attacked/chased **by elephants?**

**Please explain the event _____________________________________________________________**

1. **Have you or do you know someone who has been compensated for elephant damages, injuries or deaths?** 🗌 Yes 🗌 No

**If yes: Who? ___________________________how much (Kes)___________________**

1. **Do you think compensation is an effective way to reduce the suffering caused by elephant damages, injuries and loss of lives to families in your village?**

🗌 Yes 🗌 No: Please explain your reasons___________________________________

1. **Have you been forced to change your crops or abandon your farm because of elephant destruction?** 🗌 Yes 🗌 No
2. **Have you or any member of your family benefited from elephant conservation in the last 12 months?** 🗌 Yes 🗌 No: Please explain__________________________
3. **Who are the current losers as a result of human-elephant interactions in Trans Mara? Please rank them with “1” being the greatest loser and “6” being the least loser.**

Group ranches/ Management committees 🗌

Mara conservancy 🗌

Local community 🗌

Kenya Wildlife Service 🗌

Trans Mara county Council 🗌

Other (Please specify) 🗌

1. **Which gender group is most affected as a result of human-elephant interactions in Trans Mara? Please rank them with “1” being the most affected and “6” being the least affected.**

Male adults 🗌

Female adults 🗌

Young males 🗌

Young females 🗌

School Going Children (Boys and Girls) 🗌

Other (Please specify) 🗌

**9. ATTITUDES AND PERCEPTIONS**

1. **I am going to read multiple statements. For each statement, please tell me if you:** Strongly Agree; Agree; Uncertain/Neutral; Disagree Strongly Disagree.

| **Statement** | | **Strongly Agree** | **Agree** | **Uncertain/Neutral** | **Disagree** | **Strongly Disagree** |
| --- | --- | --- | --- | --- | --- | --- |
| 63.1 | Elephants support tourism that brings revenue to the community |  |  |  |  |  |
| 63.2 | Elephants support tourism that brings jobs to the local residents |  |  |  |  |  |
| 63.3 | Tourist lodges in TM and MMNR have created business opportunities for the local community |  |  |  |  |  |
| 63.4 | MMNR and conservancies have brought positive changes to the community |  |  |  |  |  |
| 63.5 | MMNR and Conservancies have contributed to education in the village |  |  |  |  |  |
| 63.6 | MMNR and conservancies have caused conflicts among local villagers |  |  |  |  |  |
| 63.7 | MMNR and conservancies do not benefit anyone in the village |  |  |  |  |  |
| 63.8 | Elephants have become a problem to the community |  |  |  |  |  |
| 63.7 | I like elephants |  |  |  |  |  |
| 63.10 | Elephants are responsible for more damage than they are worth |  |  |  |  |  |
| 63.11 | MMNR and conservancies do not protect elephants |  |  |  |  |  |
| 63.12 | I live better because of the conservancies |  |  |  |  |  |
| 63.13 | Tourist lodges are good for the village |  |  |  |  |  |
| 63.14 | I do not support the work of the conservancies |  |  |  |  |  |
| 63.15 | There are too many elephants |  |  |  |  |  |
| 63.16 | I would be happier if the conservancies were not there |  |  |  |  |  |

**--END--**

**THANK YOU**
