# Supplementary material for: Measuring the conservation attitudes of local communities towards the African elephant Loxodonta africana, a flagship species in the Mara ecosystem
Source: PLoS One. 2021 Jun 23;16(6):e0253234. doi: 10.1371/journal.pone.0253234 (PMC8221505; doi:10.1371/journal.pone.0253234)
Supplement: Attachment — Submitted filename: Response to Reviewers_2021.docx [file pone.0253234.s002.docx]

Dear editor and reviewers

PONE-D-21-07081

Measuring the Conservation Attitudes of Local Communities Towards the African Elephant Loxodonta africana, Flagship Species in the Mara Ecosystem

We would like to thank you for providing the reviewers’ comments which we have found to be very helpful. We thank the reviewers for their critique of the manuscript. In response, we have considered all the reviewers’ comments and suggestions systematically in the table below. In response to the specific issues raised by the editor, we have replaced figure 1 with a more detailed figure depicting details of the study are and the status of HEC to provided further clarity on our analysis and results. We have update the “materials and Methods” section to account for the consent and ethical considerations in the study and research process. Finally, we have also included the “tables” as part of the “manuscript” as directed. We have made the necessary changes in “track change” mode in the 'Revised Manuscript with Track Changes' word document. We have a separate clean “manuscript” without the track changes attached as well. In addition, we have attached a rebuttal letter “Response to reviewers” which responds specific and general reviewers’ comments and/suggestions point by point including new line numbers in the revised manuscript with track changes.

**Response to Reviewers Comments**

**Reviewer 1**

| **Line number** | **Comments** | **New line number** | **Response** |
| --- | --- | --- | --- |
| N/A | Describe salient features of the five administrative divisions with special reference to natural resources availability, demography etc. for better understanding of the situation and study area. | 138-158 | This has been addressed in the revised manuscript |
| N/A | For questionnaire survey 376 households were selected for household interviews. Authors should explain adequacy of the sample size for a desired level of precision. Further, the authors should mention the number of households surveyed in different divisions of TM and justify the number of households surveyed in the divisions. | 170-179 | This has been addressed in the revised manuscript |
| 167-169 | Authors should explain the criteria of categorization of Attitude Indices Scores into ‘negative’, ‘neutral’ and ‘positive’ attitude for clarity to readers. |  |  |
| N/A | Authors are suggested to consider– ‘distance of household / community settlement from the boundary of protected area’ as a variable in the model and see if it improves the model. | N/A | Whereas the authors acknowledge the value of this kind of analysis, we are constrained by the fact that our data collection did not include this as variable and hence we do not have the relevant data to run this analysis. However, we are persuaded that some of the current variables, especially the location of residence data (in this case the administrative division) could shed some clear light into the possible outcomes when distance is used. For example, in both EAI and MAI regression analyses, we establish that residing in areas “closer” to the park boundary (Kirindon and Lolgorian) resulted in unfavourable attitudes toward elephants and MMNR. Meanwhile, residing “far” away from the park boundary (Kilgoris and Pirrar) resulted in favourable attitudes towards elephants and MMNR (Lines 309-317).  Our discussion of the results also provides further credence to the possible outcome when distance is added to the analysis, thus “*the location of residence, in this case, the administrative division had a significant effect on the conservation attitudes. People living in Kilgoris and Pirrar divisions, which are far away from the park boundary showed favourable conservation attitudes. The two divisions are located approximately 30km to the west of the MMNR*” (Lines 419-426) |
| N/A | Have the authors worked out ‘community-forest/park’ interaction in terms of communities’ dependence on the forest areas for Provisioning Services? Direct benefits from natural habitats could be an important determinant of people’s attitudes |  | The ecosystem services derived from forests by local communities is very well acknowledged. This is undoubtedly the case with forests in TM which are open access and are predominantly under the control of group ranches as alluded to by Sitati 2003, Kipkoech et al., 2011, Miyuki & Njega 2015 and Poole *et al.* 2016. However, the scope of the present study did not capture this. Our study set out to look at the Protected area (in this case the MMNR) and the interactions between the communities and the elephants originating from the reserve. Establishing the services provided by the forests in the study area is elaborate and would have made the present study overly broad. We have therefore, only made mention of the facts thus*: Forests play an important socio-cultural role amongst the Maasai especially the provision of shade for council meetings of Maasai elders, provision of fuel, medicine, timber and shafts for spears and arrows, walking sticks, charcoal and fencing posts. In addition, they have provided a key dry season dispersal area and grazing ground for both livestock and wildlife” (*Lines 143-147). |
| N/A | The authors have collected in-depth information on attributes representing socio-economic status of studied  households. It is suggested to present key socio-economic attributes in a table and explain them aptly to improve readability and better understanding of these drivers of conservation attitude. | 254-266 | Addressed in the revised manuscript |
| 240-246 | ‘Human wellbeing’ has not been explained in the manuscript but in the Results section association of human  wellbeing and conservation attitudes have been presented (page no. 6, line nos. 240-246). The authors have  mentioned ‘higher wellbeing scores’ (line no. 246), but how these scores have been arrived at are not described in the manuscript. Secondly, in the same section 0.542** and 0.449**have been shown as a parameter value, but it is not clear what these values are? |  | We recognise the reviewer’s concern with this section and agree that indeed the manuscript has not dwelt much on the issue of “wellbeing” and how it aligns with the current analysis. In our review, we realised that the discussion around wellbeing was presented in this manuscript prematurely as it is being dealt with in a separate paper under preparation. We therefore, made a decision to omit it from the present paper with a view that doing so did not change the core of the discussion of the paper. |
| 264-265 | Have the authors studies issues related to access and equitable distribution of benefits? (page no.6, line no. 264-265). In absence of any supporting data, it seems to be speculative. | 326-334 | The present study did not evaluate the existing benefit sharing scheme between the MMNR and the local communities. But the existing literature now cited provide adequate evidence on the performance and challenges with the current benefit sharing scheme in TM. |
|  | Human-wildlife interaction substantially influences attitude of people; thus it would be good if the authors present the HEC scenario in different divisions of TM. | 157-163 | We agree with the reviewers on this and have added a figure (Figure 1) depicting the spatial distribution of HEC in TM based on the same data but in a specific paper focusing on HEC in TM by the same authors. |
|  | Page No. 9, para. 3: It is clear that opportunities are availed mainly by a select group of people in the society, and the unfavourable conservation attitude of younger generation is due to lesser opportunities and is a reflection of frustration of the youth. Do these factors also get reflected in unfavourable attitude towards governance and society at large, or these are limited only to conservation? The authors should throw light on this aspect. |  | The observed attitudes are not limited to conservation activities but could be seen in the other areas of community-government-private (Public-Private sector) interactions. A clear example of such attitudes could be observed in the government’s collaborative initiatives to introduce boarding facilities in primary schools in the district to increase pupil enrolment and retention under the universal Free Primary Education (FPE) framework. Despite the good intentions, studies, such as by Sifuna (1991) in the regions reported low uptake by the pastoralist communities based purely on cultural and economic considerations. |

**Reviewer 2**

| **Line number** | **Comments** | **New line number** | **Response** |
| --- | --- | --- | --- |
|  | The abstract has to be more precise. |  | This has been addressed |
|  | When was the study conducted? Mention in “materials and methods” section. | 125 | This is now indicated |
| 179 | Line no. 179: The number of dummy variables to be used is one less than the total number of categories. In order to simplify, the divisions can be categorized into two zones, a) zone close to MMNR (Lolgorian, Kirindon) and b) zone far from MMNR (Kilgoris, Keyian and Pirrar). Thus two dummy variables can be used for 5 divisions (suggestions are based solely on the map provided). Hence the authors are advised to rerun the regression. |  | Whereas we are not clear on the observation made by the reviewer here, we draw our response based on the basis of this observation thus “*suggestions are based solely on the map provided*”. Since the analysis was primarily statistical and not spatial, we established in lines 202-206 that the division suffered from collinearity and multicollinearity issues. Therefore, merging the divisions based on spatial layout could have obscured this attribute and, we are convinced this would have led to a faulty statistical outcome. |
|  | The attitude towards elephants (Table no. 1) is broadly tourism based. What about the positive role of the species? in the forest ecosystem? Do the locals value the existence of the mammal? These questions were not asked. Conservation of the species should not be solely based on tourism returns. Therefore, a change in the title is suggested. The title can be modified into “Exploring the Socio-Economic Aspect of Conservation Attitude in Different. Ethnic Groups towards the African Elephant in the Mara Ecosystem”. |  | We acknowledged the reviewer’s observations. But we are not convinced that the EAI is “broadly tourism based”. This is because, the index has four neutral statements, two of which make direct reference to “tourism and lodges” while the other two make reference to “damage vs worth (which is conceived to encompasses both economic/social/cultural) and “become a problem”, which encompasses conflict and losses attributed to the animals. This alone, we believe cannot justify change of the title as this will obscure the principal aim of this paper, which was not to simply measure the attitudes but also demonstrate the value of using indices to facilitate measurement, which is a deviation from the traditional attitudinal studies. |
|  | The authors can include suggestions like preservation and documentation of traditional knowledge related to wildlife, creation of more jobs connecting MMNR with the locals, proactive government in providing adequate and immediate compensation for damages, alternative livelihoods involving non-conversion of forests, involvement of locals in rescue operations of injured elephants or orphan calves as well as building task force for protection of wildlife. The young generation must be educated how to value the Masai Mara ecosystem. In case of existing agricultural lands, the farmers can be taught to safe-guard their crops using latest techniques (location-specific solutions such as bee-fencing). Adaptation and tolerance are the keys to co-existence. | 537-544 | These suggestions have been incorporated in the revised manuscript appropriately. |
| 467-468 | Line nos. 467-468: The term “battle” can be more sensitively replaced by the ecological term “competition”. The last sentence is to be omitted for not generating a negative/destructive attitude towards the species. | 536 | This has been addressed in the revised manuscript |
